# Faith Leaders’ Perspectives on Involvement in HIV Prevention for Urban Black Youth in New Jersey, USA

**DOI:** 10.3390/rel15070862

**Published:** 2024-07-17

**Authors:** Ijeoma Opara, Kimberly Pierre, Cora Gabriel, Kristina Cross, Carolanne M. L. Clark, Jaleah D. Rutledge

**Affiliations:** 1 Department of Social & Behavioral Sciences, Yale School of Public Health, Yale University, New Haven, CT 06520, USA; 2 Irvington Department of Health and Senior Services, Irvington, NJ 07111, USA; 3 Centers for Disease Control & Prevention, Atlanta, GA 30333, USA; 4 School of Social Welfare, Stony Brook University, Stony Brook, NY 11794, USA

**Keywords:** HIV prevention, black youth, community HIV prevention

## Abstract

This qualitative study takes place in an urban community that has high rates of HIV among Black youth. Six faith leaders were interviewed (five identified as Christian and one identified as Muslim). Three major themes arose from the interviews, including (1) the role of sex and HIV; (2) hindrances to sexual health conversations with youth; and (3) considering religious principles to prevent HIV in Black youth. Findings from this study can be used to inform an HIV-prevention curriculum for Black youth who identify strongly with their religion and spirituality and live in high HIV-risk communities.

## 1. Introduction

About one million people in the U.S. have been diagnosed with HIV with Black and Hispanic/Latinx people being disproportionately affected (Centers for Disease Control and Prevention [CDC] 2023). Youth between the ages of 13 and 24 represent 19% of new HIV cases (Centers for Disease Control and Prevention [CDC] 2024). These populations often face a range of individual (e.g., knowledge, attitudes, and behavior) and systemic challenges (e.g., racism, sexism, and access to care) that drive HIV inequities within their communities (Filippone et al. 2023). Some of the literature specifically emphasizes how the social determinants of health, which are “the conditions in which people are born, grow, live, work, and age, including the health system” are inextricably linked to racial disparities in HIV/AIDS (Robinson and Moodie-Mills 2012, p. 2).

Black youth are at greatest risk of contracting HIV, have low adherence to treatment, and have more limited access to HIV testing than other ethnic minority youth in the United States (Boyd et al. 2018). Particularly among youth living in urban communities, they often have less access to HIV-prevention resources and culturally appropriate educational programming (Lardier et al. 2021; Opara et al. 2021). Within New Jersey, HIV/AIDS continues to be an epidemic (New Jersey Department of Health 2020). Currently, New Jersey ranks fourth in the nation for overall cumulative HIV cases among adults/adolescents and has the fifth-highest rate of HIV among children (New Jersey Department of Health 2020). HIV is a significant threat in the Black community especially as Black people account for 52% of persons living with HIV, despite declines in infection and reported deaths (New Jersey Department of Health 2020). The availability of community leaders and resources within the community can serve as a strategy for increasing knowledge and influencing positive behaviors in vulnerable, more susceptible populations. Historically, leaders of faith organizations have played a significant role in delivering health and social services in developed countries (Griffith et al. 2010); within the United States, an estimated 36% (118 million) of adults have reported that they regularly attend religious services (Koh and Coles 2019). Faith organizations and leaders can play a role in providing sustainable interventions in HIV prevention due to their diverse presence and extensive reach, especially within the Black community (Stewart 2015). For example, Wingood et al. (2011) developed intervention using SISTA (Sisters Informing Sisters on Topics about AIDS), a Centers for Disease Control and Prevention defined evidence-based HIV-prevention intervention for Black women (Wingood and DiClemente 2001), and adapted it to include a religious perspective (Wingood et al. 2013). Results from a small feasibility study of the adapted intervention found that Black women were favorable to the intervention and it improved HIV education significantly through a faith perspective (Wingood et al. 2011).

There has been strong evidence of the positive health impact of involvement in faith organizations among Black Americans due to spirituality and religiosity (Ransome et al. 2018). Despite faith organizations such as churches being a noted pillar for the Black faith community, little is known of the perception of faith leaders’ role in ending the HIV epidemic among Black youth. Within the United States, HIV has disproportionately affected communities of color, including Black people (Bradley et al. 2018; Coleman et al. 2016; Pellowski et al. 2013). Black churches engaging in health promotion interventions present opportunities for positive health outcomes and behaviors but also raise challenges for public health researchers in reducing HIV risk specifically for those communities who are at greatest risk (Stewart et al. 2013).

Black churches and other faith settings (e.g., mosques) have been criticized by some as contributing to the stigmatization of people living with HIV and being lacking in involvement in community efforts around HIV prevention (Berkley-Patton et al. 2013; Bradley et al. 2018). HIV-related stigma within religious settings continues to present a substantial challenge to HIV prevention which can adversely affect strategies that can derail the participation of individuals in HIV education, prevention, testing, and treatment efforts (Bradley et al. 2018; Coleman et al. 2016). With collaborative efforts between faith organizations and public health services, better understanding and access to prevention interventions can be combined with both faith and secular, evidence-based concepts to present to affiliated religious members. Faith interventions that tailor interventions can be an effective tool to address HIV-related stigma in the Black community and other communities of color at risk in the United States (Coleman et al. 2016).

## 2. Purpose of Study

Utilizing semi-structured interviews with prominent faith leaders in an urban community in New Jersey, this study aims to explore faith leaders’ attitudes and perceptions about HIV-risk and prevention education for youth. Findings from this research can contribute to the development of faith-based HIV education, prevention, and stigma reduction interventions in urban areas that primarily serve youth of color.

## 3. Methods

### 3.1. Community Profile

The study takes place in Paterson, New Jersey, which is one of the top ten cities accounting for 65% percent of the state’s African Americans/Black living with HIV/AIDS (New Jersey Department of Health 2018). Forty-two percent of reported diagnoses of Paterson residents (including adults) were as a result of injection drug use (New Jersey Department of Health 2018). Paterson is one of the most diverse cities in New Jersey, with 57.7% identifying as Hispanic, 34.7% identifying as African American/Black, followed by 31.7% identifying as White (United States Census Bureau 2020). Approximately 43.3% residents living in this city are foreign born (United States Census Bureau 2020) and approximately 29.1% live below the poverty line, with a median household income of $33,000 yearly compared to 10.4% and $71,180 median income for the entire state, respectively (United States Census Bureau 2020). The city is one of the poorest cities in New Jersey, with a median income that is among the lowest in the state (United States Census Bureau 2020). The city’s child poverty rate is 41%, which is higher than New Jersey’s rate of children in poverty, which is 16% (Advocates for New Jersey Kids Count 2015).

### 3.2. Recruitment of Faith Leaders

The study was approved by the Montclair State University Institutional Review Board as a part of a larger federal funded project that sought to mobilize community members to create more awareness around youth HIV prevention. Snowball sampling (Johnson 2014), was used to recruit six African American pastors in Paterson, New Jersey into an HIV-stigma research study in faith settings. See Table 1 for participant demographic characteristics. The research team utilized community contacts to initiate recruitment of leaders in the community and asked leaders to share with their networks. Several pastors reached out to the first author and provided names of leaders who were interested in the study. Pre-existing relationships with community and university organizations, HIV-advocacy groups, and nonprofit organizations helped with the recruitment of leaders. The study was a part of a larger study funded by the Minority AIDS Initiative, which was reviewed and approved by the Montclair State University Institutional Review Board.

### 3.3. Procedures

Faith leaders were contacted by the first author to be a part of the interview to ensure that they were eligible to take part in the study. Inclusion criteria for leaders included: being a senior pastor or leader in a church or mosque that has a youth ministry, and presiding over a predominately Black congregation in Paterson, New Jersey. Once the leaders agreed, and met the eligibility criteria, an interview date was scheduled. All interviews were held in person in private, closed rooms in the leaders’ organizations (five churches and one mosque) and were recorded for transcription purposes. Informed written consent was obtained from each participant. Semi-structured interviews were utilized to encourage informants to speak freely and to minimize the elicitation of the interviewers’ preconceived ideas. In total, six interviews were conducted, and each lasted for approximately 45 min–60 min.

### 3.4. Data Analysis

Interviews were transcribed verbatim by a master’s level research assistant. Transcripts were listened to and verified by the first author to check for accuracy. Analysis of interview data involved five coders. Five coders read and re-read each transcript weekly, coded the transcript and met as a group to discuss the findings while the second author led the weekly discussions. The first author, who interviewed all of the participants, met with the second author weekly to discuss any discrepancies and themes that arose in the group of coders. Analysis proceeded inductively through the identification of recurring themes and patterns in transcripts, field notes, and analytic memos. Meaningful analytical units were then developed by using a coding scheme that was informed by dominant themes in the data. These topics were then divided into several subtopics based on recurring themes within the larger topics, allowing for more in-depth analysis and complex understanding and interpretation of each particular theme. Each theme and subtheme was assigned a code, and the codes were compiled in a codebook. The coders then clarified the codes’ definitions and ensured that all codes fitted into a structure with meaningful and salient inter-relations and distinctions among them. Open and axial coding were then used simultaneously as data were delineated into concepts. Subsequently, the relationship between concepts and categories was analyzed. Quality checks were undertaken to ensure high inter- and intra-coder reliability among coders. After attaining inter-rater reliability of 95%, defined as the number of agreements divided by the number of agreements plus disagreements, authors coded all data. The resulting data were utilized to examine the specific research questions guiding the proposed study.

## 4. Results

Six faith leaders in Paterson, New Jersey were asked a series of questions in their interviews about the structure of their congregation, their efforts within the community, and whether sensitive topics such as sexual health, HIV prevention, and mental health are addressed (see Appendix A). Three major themes arose from the interviews including (1) the role of sex and HIV; (2) hindrances to sexual health conversations with youth; and (3) considering religious principles to prevent HIV in Black youth.

### 4.1. The Role of Sex and HIV

Five out of six participants mentioned that they believe the HIV epidemic is caused mainly by “promiscuousness”, “prostitution”, “sex before marriage”, and “homosexuality”. All of the leaders mentioned the high rates of sex work, which they referred to as “prostitution”, in their town. One of the leaders emphasized, “the women that are prostituting, they catch it [HIV] and they spread it to others”.

There were varying perspectives about the role of faith and religion within their community in responding to the HIV epidemic. All six leaders agreed that it is important to address; however, some felt that it would be an uncomfortable topic to discuss in their congregations. One leader, a Christian pastor, conveyed:

“I think people [of faith] don’t see that as a conversation that needs to happen in a church. . .the Bible says no sex before marriage. Now if you feel that’s what you got to do, then you need to consult resources in your community where they can provide you the support for that”.

Conversely another faith leader, who has hosted events during the national week of prayer for the healing of AIDS in the past, stated his congregation uses an AIDS quilt “that we dedicated years ago. . .in memory [of] people [that] died of HIV. . .as a way to celebrate their lives. . .to try to take away the stigma from people who are affected or infected but also for their family”. His views of the role of the church and faith in their community differed from the other participants mainly because of his training and experience as he is a nationally known advocate of HIV/AIDS prevention in the church. Two out of six of the leaders believed that there needed to be more work done within faith-based communities to change the perspective of topics such as sex and HIV in order to truly have impactful conversations about prevention.

All participants in the study attributed the high prevalence of HIV in their community to a lack of religious values in the nation and also a lack of understanding of HIV education. As one leader mentioned, contributing factors to this epidemic in this particular city could be due to “lack of education and also probably have to do with access to protection”. While participants in the study acknowledged that changes in society should lead to more direct conversations, they agreed that this may be uncomfortable given the perceived level of intrusiveness.

Within the context of Black youth, leaders felt that it was a responsibility of schools in their community to discuss HIV/AIDS and safe sex practices with youth; “I don’t believe that schools are educating our kids on this issue”, one leader stated. Many participants agreed that education is crucial for initiating and sustaining positive change around the HIV epidemic. Comparing history lessons with social impacts on the community’s health, one faith leader stated, “We need to re-educate ourselves, not so much about George Washington or Abraham Lincoln. But we need to get the modern technology that’s going to take us some place”. Throughout the years, society has conformed in various ways and to address youth, it’s important for authority figures to find ways to relate to them, in the hope of having lessons resonate. Finally, one leader simply stated, “Knowledge is powerful. . . Education is powerful”. The conciseness of this statement highlights the importance of being informed, especially around topics such as HIV and sex, that are typically considered taboo. As the study leaders grappled with the reality of youth engaging in sexual activity, they acknowledged the need to address this issue. However, they noted negative connotations associated with it hinders progression.

### 4.2. Hindrances to Sexual Health Conversations with Youth

All leaders acknowledged that it can be difficult to discuss sex and HIV with youth in their spaces due to the judgmental nature of church leaders; one participant mentioned, “the church can often come across as. . . judgmental”. For example, one faith leader shared that “most of the education [his congregation does] is around [abstinence]”. Another leader, one of the two female pastors in the sample, discussed the difficulty in working with youth who are believers and non-believers on this topic because of the way religion has been painted by the media:

I think the media has painted Christianity as a bigoted belief. . .You know, very narrow-minded type of belief system. Which is crazy. . .Because if you look at the life of Jesus. . .He was with all the people who were the outcast. So how do we get to become this exclusive club of people who don’t want anything to do with people who don’t think, and look like us? It’s just crazy. . .we lost that culture war so now. . .the church has to go to the community.

When responding to the same question, another pastor felt “some church[es], some Christian people live in a vacuum now like this stuff doesn’t go on. It goes on”. He later stated “I think that our narrowness is a barrier. . .too many people are trying to make people holy. . .But people, ‘holy’ makes people ‘whole’”.

Another faith leader shared that they weren’t sure the lead pastor of their congregation would be open to having an HIV/AIDS program in the church: “Most people who go to church . . . don’t really want to hear about health. They don’t want to hear about AIDS. . . They don’t want to hear about substance abuse until it affects their family”.

Lastly, one participant shared their powerful perspective about the Black Church’s efforts in prevention:

“I think that the Black Church, in particular, has to challenge itself to be more proactive and have inroads into the areas that hurt out people so we can help our people. . . I think that’s what we’re missing. We are in a. . . silo trying to pretend this stuff doesn’t exist. . . [The Black Church needs] to be embraced and empowered and strengthened and affirmed so that they can help other people, and they live in silence because they’re afraid to speak out”.

### 4.3. Considering Religious Principles to Prevent HIV in Black Youth

While there was some disagreement as to whether HIV was an issue in their community, faith leaders in the study were adamant about relying on Biblical scriptures which can aid in HIV-prevention efforts. One leader mentioned:

“The Bible was written for a reason. It was written to give us a model for how to live our lives. . .We don’t believe in sex before marriage. We don’t believe in cohabitation. We don’t believe in things that lead to outcomes that are unhealthy. . . [In regard to soul ties], what people don’t realize is when they have sex, it’s spiritual. We believe [having multiple sexual partners] opens you up to thought processes. . . and just not being centered as a person because you’ve taken on all that stuff that the other person was thinking and dealing [with] plus your own. And if you got multiple partners, you [could] become confused. . .”.

The idea of the body being a “temple” was also an emphasized perspective of prevention:

“If my body is a temple, that is the habitation for God the Holy Spirit, then really, do I want to have a lot of mixed-up stuff going on? . . . If you begin to recognize that God created each and every one of us for a purpose, and if we get involved in some of these behaviors that are not healthy, [then] it derails the ultimate purpose for which God created us. . . We’re not honoring God when we participate in those kinds of things”.

Additionally, another faith leader stated that faith or religion has everything to do with the prevention of HIV/AIDS:

“When you have conviction and . . . give yourself over to a higher force or a higher power, your willpower, your soul becomes strengthened and embodied with the enlightenment to overcome the Satanic things that take you away from the higher power”.

Another faith leader disclosed a similar idea that describes the role of knowing that one is a new creation of Christ:

“. . .Really get that in their heart. They are going to think twice; and if you keep them engaged. . ., they’re not apt to go out and get on drugs or. . . be promiscuous. . . and are accountable to God. . . get the youth to know God on a personal level. You gotta get them to the point of loving themselves”.

Another leader mentioned:

“The Bible says no sex before marriage. Now, if you feel that you have to have sex, and that’s what you got to do, then you need to consult resources in your community where they can provide you with the support for that because, if you’re going to do it. . ..we don’t agree”. When asked if leaders were aware of resources for their members, five out of six participants admitted they were not aware of where to send their members to. The only Imam in the study mentioned, “Honestly, I don’t even know where to send them to. If someone came to me saying they had HIV or asked how do I prevent myself from getting HIV? Do I wear condoms? I would encourage them to seek Allah but then not sure if I should be doing more”.

Leaders in the study were divided on how to effectively discuss HIV prevention without compromising their values. Three faith leaders admitted that providing youth with contraception, for example, is perceived as “just giving [congregation members] permission to go have sex”.

This faith leader connected this idea to the Black Church’s involvement in combating racism:

“I think that. . . is a rule that the Black Church has played in America. . . forever.

Because of institutionalized racism. . . that we’ve dealt with. . ., it’s been the faith of people that said, ‘OK. This is our reality, but it’s not all there is’. I think it’s the responsibility issue. . . We have to teach people how to be responsible”.

Overall, while leaders overwhelmingly believed that faith and spirituality are central to preventing HIV, they acknowledged how uncomfortable it may be to have these conversations within the church.

Among all the participants, there was similar advice which encouraged research or prevention teams to not forget about the role of religion in prevention for youth. While a majority of the participants were against discussions of consensual sex before marriage, one leader expressed the importance of being honest with the church even if they, as leaders, are not receptive. “My message to researchers is don’t cut corners or have misdirection. . . because it’s very important to us that all the facts be there, whether we like it or not. . . Stand up for the truth so that we can end the virus”.

## 5. Discussion

This qualitative study explored faith leaders’ perspectives on HIV prevention in an urban community. Faith-based leaders in this study discussed the role of sex, hinderances to sexual health conversations with youth, and the use of religious principles for HIV prevention in Black youth. Each of these themes reveal unique insights regarding faith leaders’ attitudes and perceptions regarding HIV prevention. Consistent with the literature, Black faith institutions are aware of the health disparities such as HIV, that are negatively impacting their community and congregation (Moscati and Mezuk 2014). An awareness of HIV disparities is an important initial step in laying the foundation for HIV education and prevention efforts within faith organizations. Unfortunately, the participants focused on HIV as an individual-level issue and not a systematic or structural issue. This finding reveals the potential for major challenges when addressing the root causes of HIV. A limited understanding of the role of systemic and structural factors in HIV transmission may reflect a barrier to HIV prevention within religious institutions. Findings from a similar study suggest that faith leaders may have a more effective impact by engaging in discussions around HIV that focus on structural and systemic issues as opposed to individual discussions of sexuality (Nunn et al. 2013). Therefore, it may be more constructive for faith leaders to receive and prioritize education around the social determinants of health and HIV disparities.

Overall, the leaders believed that grounding youth in spirituality and religion can contribute to a reduction in HIV. All participants expressed a religious and moral imperative to assist youth in sexual health issues (Hidalgo et al. 2019; Woods-Jaeger et al. 2014). At the same time, participants also believed that schools hold responsibility for educating youth as well. Although the leaders acknowledged the importance of discussing sex and HIV with youth, they also recognized various barriers they may face in navigating these conversations that may not exist in schools. The participants related their religious doctrine and recited from sacred texts (e.g., the Bible and Quran) to state the importance of serving others particularly in prevention of HIV and also substance use. While some faith leaders expressed hesitation or concern with how the information could be relayed, all participants were open to having programs implemented in their congregations for HIV prevention, hosting HIV awareness and prevention events, or having a health service navigator, as long as they included a religious perspective. A study conducted by Woods-Jaeger et al. (2014) found church values and principles can be in line with health messaging and provide a foundation for effective HIV-prevention programs for Black youth. Among the six faith leaders in the study, only one participant acknowledged receiving formal HIV education and prevention training. In addition, nearly all of the participants admitted to a lack of knowledge around resources for members of their faith community who may admit to being HIV positive. Based on these two findings, it may be important for public health practitioners to focus efforts on providing formal HIV education and prevention training for faith leaders, considering that research suggests churches are effective sites for the delivery and implementation of health programming, especially for Black people (Francis and Liverpool 2009; Wooster et al. 2011).

Every participant acknowledged the potential difficulties in talking to youth about sex and HIV. This finding is consistent with the literature on faith leaders, religious institutions, and sexual health education (Golman et al. 2021; Haglund and Fehring 2010). For example, in another qualitative study on the perspective of faith-based leaders’ regarding HIV prevention in the Black church, faith leaders described calling on experts and health professionals to help initiate the conversation around HIV (Bryant-Davis et al. 2016). This strategy may be a promising option for a way to discuss sex and HIV in religious spaces. Regarding safe sex practices, some of the leaders noted, consistent with the literature (Lightfoot et al. 2014; McRoberts 2003), that they focused their HIV-prevention initiatives through the lens of abstinence. Conversely some participants mentioned openness to educating adolescents about safe sex and promoting condom use. Other studies that have examined the role of faith leaders and HIV prevention, underscore the importance of understanding the extent to which churches are to address HIV within their congregations. This understanding is a necessary precursor to designing interventions in religious settings. Griffith et al. (2010) report that a major barrier to an effective response by the Black Church is negative religious and moral attitudes about behaviors associated with HIV transmission, such as sexual behavior. However, with collaboration, training, and technical assistance, faith institutions can begin to restore the challenge of connecting HIV prevention with their religious and spiritual missions. Principal to this approach is respecting the values and beliefs of faith leaders and treating HIV as a health issue, along with a sexual and moral issue (Griffith et al. 2010).

## 6. Limitations

Although our research contributes significantly to the limited literature, our study has a few limitations. It was important to utilize a qualitative approach to effectively capture the shared lived experiences of participants. Our small sample size, which centered on one community, and lack of religious diversity may make it difficult to generalize findings. However, the goal of qualitative research is not to generalize, but to understand the unique experiences of participants. The findings of this study can contribute to further exploratory studies and literature pertaining to this work. Future research should examine faith leaders who identify with other religious groups, such as Islam, Buddhism, Judaism, Hinduism, Christianity, etc.

## 7. Conclusions

These study findings, while modest, provide important implications for HIV-prevention education within faith communities. To effectively contribute to addressing barriers to HIV prevention and treatment, community-based efforts require local leaders to acknowledge the severity of the continuing epidemic among Black youth and help reduce the spread of HIV. Working collaboratively with religious leaders in the Black community, prevention researchers can begin to provide messaging around HIV etiology and prevention with youth. With proper training, education, and technical assistance, faith organizations can be equipped to provide HIV-prevention education. The findings point to a need for faith leaders to evolve beyond an individual-level perspective and confront homophobia and sex shaming, which are also important in faith organizations, to create an environment for effectively discussing HIV with youth. For the Black community specifically, collaborations between faith organizations, public health agencies, and other community stakeholders is essential for combating HIV.

Utilizing a strengths-based community-service-oriented model in conjunction with faith organizations’ resources, connections, and impact could prove more effective in HIV prevention (Bluthenthal et al. 2012; Stewart 2015). Long-standing collaborations between academics, prevention specialists, and faith communities in the ever-changing landscape of the HIV epidemic could provide sustainable HIV-prevention programs in arears with populations that are impacted disproportionally (Nunn et al. 2013). Programs that target the current level of openness of faith leaders and the pragmatic capacity of faith institutions could be a starting point to collaborating with faith communities (Nunn et al. 2013; Stewart 2015). Further studies addressing HIV-prevention programs within Black faith communities from an ecological, strengths-based, social justice perspective could be leveraged to shift away from an individual-level perspective of HIV prevention.

## Figures and Tables

**Table 1.** Participant Demographic Characteristics.

Participant	Age Group	Religious Affiliation	Gender	Race	Size of Congregation	Length of Time as a Leader

Participant 1	55+	Muslim	Male	Black	50–100	4 years
Participant 2	55+	Christian	Female	Black	50–100	7 years
Participant 3	Between 45–55	Christian	Male	Black	101–250	22 years
Participant 4	55+	Christian	Female	Black	50–100	25 years
Participant 5	37–45	Christian	Male	White	50–100	10 years
Participant 6	55+	Christian	Male	Black	50–100	13 years

## Data Availability

The dataset presented in this article are not readily available because of confidentiality of the participants and technical limitations.

## Appendix A. Interview Guide

1. Tell me about any HIV/AIDS education that you have received?
  - Where can someone in your congregation go to receive quality HIV/AIDS education?
  - What about HIV/AIDS testing? What about treatment?
  - Do you know where people can go to get tested for HIV? How about HIV/AIDS treatment?
2. Tell me about any substance abuse education that you received?
  - Where can someone in your congregation go to receive quality substance abuse education?
  - What about substance abuse treatment?
3. What are the key factors that are driving the HIV/AIDS epidemic?
4. What are the key factors that are driving the substance abuse epidemic?
5. Where can someone in your church go to if they found out they were HIV positive? Or were addicted to drugs?
  - are there any ministries specifically dedicated to HIV/AIDS and/or substance abuse?
6. What role, if any, does faith or religion have in prevention?
  - Do you have any examples?
7. Does your church have a curriculum that discusses HIV/AIDS, sexual health and/or drug use?
8. Would you be open to having a HIV/AIDS and substance abuse prevention program in your church? Why or why not?
9. What would a HIV/AIDS and substance abuse prevention program in your church look like?
  - What components do you think would be important to include
  - What times (days and hours) would be best to conduct this type of program?
10. What is the most attended non-service program in your church (e.g., bible study, Sunday school)
  - does this program have a curriculum to address HIV/AIDS or substance abuse?
  - Would your congregation be open to this inclusion? If YES, how would it look like?
11. Can you give me a sense of health-related issues that has impacted your congregation? (e.g., asthma, diabetes)
  - Which age group is impacted by this?
  - What is in place to address this issue?
12. What advice would you give the research team interested in creating a prevention program your church?
13. What is your view of the state of religion and faith in the United States currently?
